# High tibial osteotomy and additive manufacture can significantly reduce the climate impact of surgically treating knee osteoarthritis

**DOI:** 10.1007/s11367-025-02473-4

**Published:** 2025-06-13

**Authors:** R. L. Anspach, H. S. Gill, V. Dhokia, R. C. Lupton

**Affiliations:** 1 Department of Mechanical Engineering, University of Bath, Claverton Down, Bath, BA2 7AY UK; 2 Institute of Sustainability and Climate Change, University of Bath, Claverton Down, Bath, BA2 7AY UK; 3 Centre for Therapeutic Innovation, University of Bath, Claverton Down, Bath, BA2 7AY UK; 4 Centre for Bioengineering & Biomedical Technologies, University of Bath, Claverton Down, Bath, BA2 7AY UK; 5 Centre for Digital, Manufacturing & Design, University of Bath, Claverton Down, Bath, BA2 7AY UK

**Keywords:** High tibial osteotomy, HTO, Unicompartmental knee replacement, UKR, Additive manufacturing, Conventional manufacturing, Life cycle assessment, Uncertainty analysis

## Abstract

**Purpose:**

This study examines the climate impact of two surgical treatments for knee osteoarthritis, unicompartmental knee replacement (UKR) and high tibial osteotomy (HTO), also comparing conventional manufacturing (CM) with additive manufacturing (AM) for HTO. Factors beyond the implants themselves are considered that depend on the manufacturing method, such as surgical instruments and guides (jig), sterilisation, transport and anesthesia using data obtained first hand from manufacturers and a hospital.

**Method:**

The relevance of the comparative results are maximised beyond a specific manufacturer’s product by including uncertainty in the foreground and background life cycle inventories to represent uncertainty and variability of process characteristics, materials, and geographical location. The analysis is carried out in Brightway 2 using Ecoinvent inventory data and impacts are calculated across 18 mid-point categories. To consider possible improvement to the environmental impact of the surgical interventions, alternative electricity and surgical guide (jig) material scenarios are considered.

**Results:**

The climate change impact of UKR, 37.9 (36.8–38.9) kg CO$_{2e}$, is highly significantly greater than that of the CM HTO, 10.7 (10.0–11.4) kg CO$_{2e}$, and AM HTO, 13.4 (13.0–13.7) kg CO$_{2e}$. The custom single-use surgical jig of the AM HTO and the use of potentially higher-carbon electricity leads to the AM HTO having an impact 1.25 (1.17–1.34) times higher than the CM HTO. But when low-carbon electricity is used and the surgical guide is made of stainless steel, this reduces to 0.78 (0.73–0.84). Initial screening of other lifecycle impact categories shows similar trends in most cases.

**Conclusions:**

This study concludes that HTO has highly significantly lower climate change impact than UKR. AM HTO has the potential to further reduce the climate impact compared to CM HTO given low-carbon energy supply and further improvements in material choice and design optimisation. Challenges include limited availability in design skill-set for optimisation and higher cost for healthcare providers compared to CM HTO, although still lower than the cost of UKR. Our study highlights policy implications: along with being a solution for early treatment and yielding improved correction accuracy compared to CM HTO, personalised AM HTO also offers environmental benefits if designed and manufactured well.

**Supplementary Information:**

The online version contains supplementary material available at 10.1007/s11367-025-02473-4.

## Introduction

Knee osteoarthritis (OA) is very common and is increasing in incidence. Knee OA is responsible for a large personal and social burden, it is amongst the leading causes of disability and is associated with excess mortality and high healthcare spending (Palazzo et al. 2016; GBD 2021 Osteoarthritis Collaborators 2023). Knee OA incidence is projected to increase 75% (GBD 2021 Osteoarthritis Collaborators 2023) by 2050 relative to current levels. It is linked to ageing and obesity (Palazzo et al. 2016; Blagojevic et al. 2010), and as the population ages together with becoming more obese, this is driving the increase in incidence and thus the demand for treatment (Patel et al. 2015). In the vast majority of cases, knee OA begins in the inside part of the knee, which is termed the medial compartment (White et al. 1991). When the damage is limited to the medial compartment, knee OA can be treated using conventionally manufactured unicompartmental knee replacement (CM UKR) or a joint preserving surgery called high tibial osteotomy (HTO) (Zhang et al. 2023). Joint replacement such as CM UKR or total knee replacement (TKR) is only recommended at the end stage of knee osteoarthritis when there is bone-on-bone contact, whereas HTO can be performed at earlier stages of the disease.

Implant manufacturers are beginning to use additive manufacturing (AM) techniques to manufacture implants (Katsuura and Qureshi 2020), particularly for personalised or custom devices. For example, a novel personalised HTO device (TOKA, Orthoscape, UK) has recently become available (Zaffagnini et al. 2022). This device utilises metal additive manufacturing techniques for the personalised surgical jig and HTO stabilisation plate. AM technologies have the capabilities to design complex components potentially saving material but at a higher cost for healthcare providers which can be offset if better outcomes and lower revision rates are achieved. However, the environmental impact of AM devices compared to conventionally manufactured devices is still unclear. With the global healthcare additive manufacturing market expected to grow by 22% from 2022 to 2030 due to the rising demand for customised medical implants (GrandViewResearch 2022) and increasing use of AM technology in orthopaedics (Meng et al. 2023), it is important to understand the impact of these trends on health sector decarbonisation targets. The UK’s National Health Service (NHS) aims to achieve a reduction of 80% in carbon emissions by 2032 (NHS 2020). To achieve this target the NHS is committed to mitigate its Scope 3 emissions, currently its medical devices being responsible for 10% of its carbon emissions, by substituting products for new technologies and innovations with improved patient outcomes and reduced impact on the climate.

The difference in environmental impacts of using AM versus CM is complex. AM processes have an environmental impact per mass of material processed that can be two orders of magnitude higher than CM processes (Kellens et al. 2017), when considering the manufacturing process alone. This observation is supported by data compiled from literature and databases by Gutowski et al. (2017) and Van Sice and Faludi (2021). Despite this, the use of AM processes can result in net savings where the material impacts are high and additive technologies can lower part mass by producing hollow and lattice structures, or mitigate the scrap generated by subtractive CM processes (Priarone et al. 2017). However, some studies report that the part mass saving due to AM was not enough to make AM the most environmentally favourable choice (Ingarao 2018). Van Sice and Faludi (2021) highlight the difficulty to derive general conclusions on the relative environmental burden of AM processes compared to non-subtractive or subtractive CM processes without considering the reference product’s specificity, and other life cycle stages which can be impacted by manufacturing techniques.

The specific energy requirement of AM was shown to vary significantly depending on the material. Selective laser melting (SLM) resulted in a specific energy requirement of 96.8 MJ per kg of chromium-nickel austenitic stainless steel deposited, calculated by Baumers et al. (2011) based on data from Kellens et al. (2011), and 471 MJ per kg of aluminium deposited (Ingarao 2018). But, it varies based on other factors such as the AM technology and the capacity utilisation (single or full bed) and layer thickness (Baumers et al. 2011). For very low part quantities the impact of CM tooling can have a large relative share in total impacts, whereas AM impact per part does not change with production volume (Faludi et al. 2017). However, if a high precision tolerance is required the additive manufactured part might still go through additional machining, in which case other non–subtractive CM processes are just as environmentally advantageous (Van Sice and Faludi 2021). Where the product consists of several complex parts, AM processes can integrate several manufacturing steps into a single step, drastically reducing the infrastructure, the number of separate manufacturing processes and transport needed to produce and assemble the product (Ford and Despeisse 2016).

The environmental benefits of AM versus CM for knee OA treatment devices therefore cannot be based on general trends but must be studied for the specific devices and materials that are used. This has been studied for one pathology, unicompartmental knee osteoarthritis. Lyons et al. (2021) carried out a life cycle assessment for AM and CM UKR devices focusing on the implant itself. Lyons et al. (2021) concluded in favour of additive manufacturing for UKR, arguing that electron beam melting uses just 22% of the material needed for CM and yields a 45% lower primary energy consumption. However, there are further factors beyond the device itself which can balance the results in favour of AM or CM processes for any given treatment. A more complete overview of impacts occurring as a result of treatment was explored for CM TKR by McGain et al. (2024), who included sterilisation, generic surgical equipment, anesthesia and pharmaceuticals, among others, in the system boundary. Rizan et al. (2023) and Delaie et al. (2023) carried out a study on conventional manufactured TKRs including impacts beyond implants. But choices around manufacturing methods were not the focus of these studies. Moreover, no studies were found investigating HTOs and comparing treatment options: early stage knee OA is suitable for treatment by HTO as well as CM UKR (Zhang et al. 2023). The novelty of this work is that it compares the environmental impact, focusing on climate change, of two treatments for knee osteoarthritis (UKR and HTO), including one that was not studied before, relative to the treatment type and manufacturing method (AM or CM) including broader implications beyond the devices themselves.

The aim of this study is to:

- Compare the overall environmental impact of CM UKR, CM HTO and AM HTO devices, including broader impacts of transport, device sterilisation, re-use, and surgery. Quantify the benefits of AM for HTO, in the context of differences between CM UKR and HTO implants.
- Investigate the major factors contributing to these impacts, and how they would alter in selected scenarios.
- Conduct an initial screening of other life cycle assessment (LCA) impact assessment categories to highlight potential burden-shifting or hotspots.

**Fig. 1:**
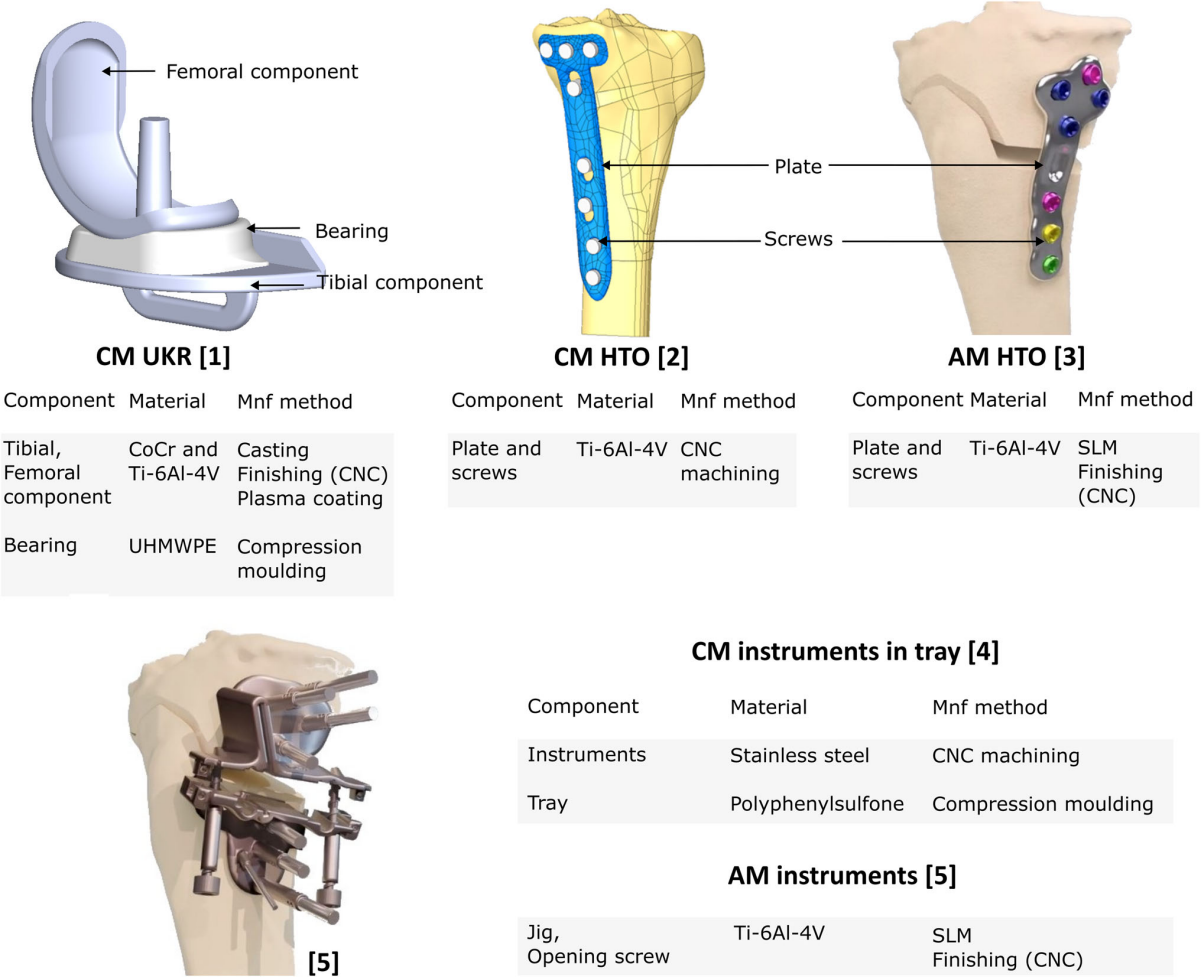
The CM UKR [1], the CM HTO [2] and AM HTO [3] devices with details on their components, materials and manufacturing methods. The instrument tools and tray are used during the implantation of the CM UKR and CM HTO devices [4] whereas the opening screws and jig [5] are used during the implantation of the AM HTO plate. Mnf, manufacturing

## Method and approach

Each subsection of the article refers to one principle of the framework defined in the standard ISO 14040:2006 (ISO14040:2006 2022).

### Goal and scope of the study

The goal of this study is to evaluate whether, in general, the use of an AM HTO results in lesser environmental harm than a CM HTO and to compare the differences in environmental impacts of knee surgery for knee OA relative to the type of the intervention (CM UKR or HTO). The goal of this study is not to quantify the impact of a specific manufacturer’s product, but to maximise the relevance of the results. Based on these starting points, inventories are defined with ranges of possible values to represent uncertainty and variability in process characteristics, materials, and geographical location. The final results therefore contain substantial uncertainty ranges, which can be compared to show whether robust conclusions about the relative impacts of the different devices and manufacturing approaches can be determined in general, or if the outcome is contingent on the specific choices made for each device. The processes and devices modelled are based on knowledge of real devices (illustrated in Fig. 1, and described below).

**Fig. 2:**
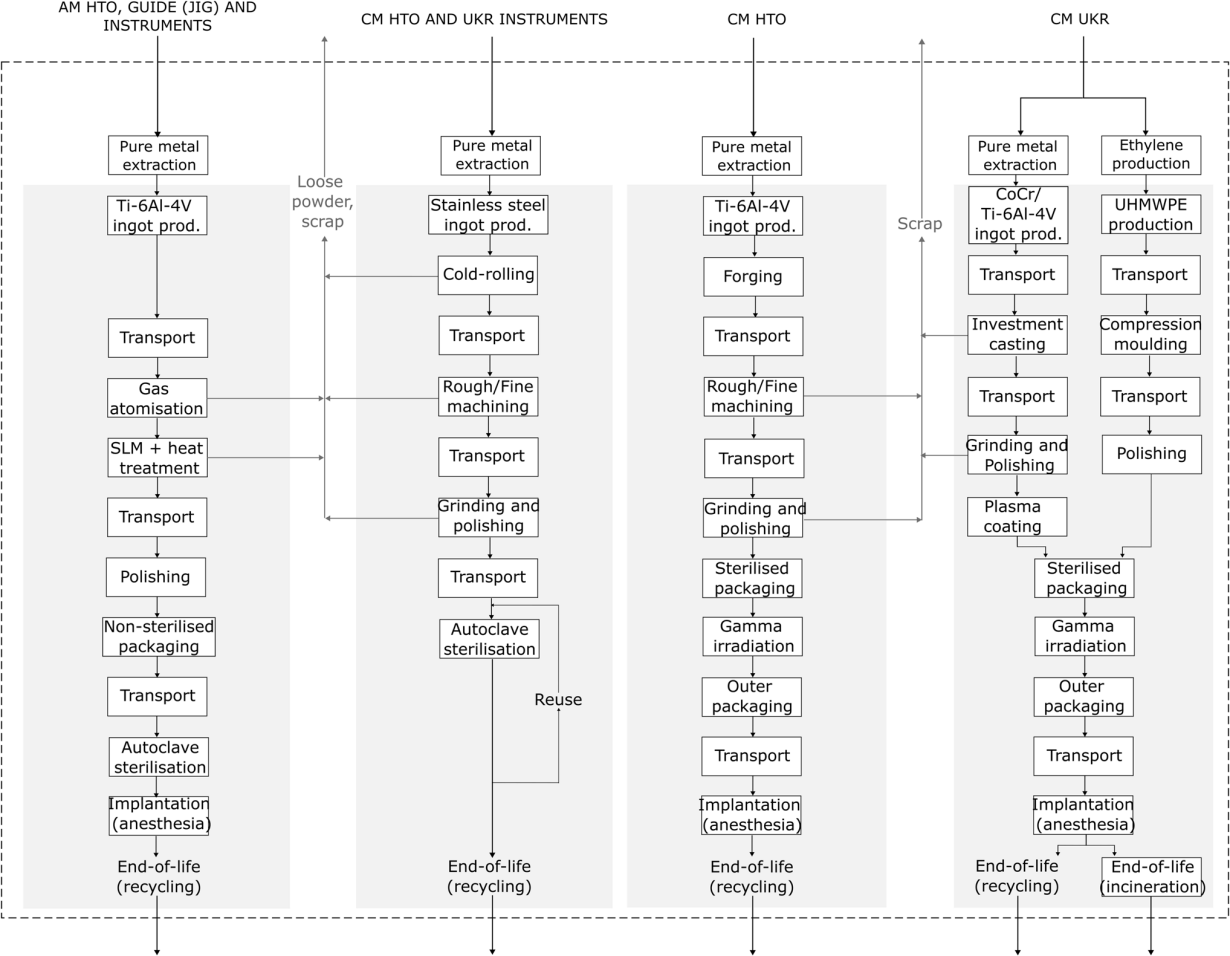
System boundary of the study from the extraction of pure metals and other base materials (ethylene) to the end-of-life of the implants and instruments. The foreground system is shaded

The component types, material and manufacturing method related to the three interventions are summarised in Fig. 1. The system boundary includes the processes from raw material production to the implantation of the parts at the hospital and their end-of-life, as shown in Fig. 2. Thus, this investigation’s scope includes the following:

- Impacts of the extraction and processing of raw materials (i.e. pure metals) required to manufacture the part including in-process material losses.
- Impacts occurring during conventional manufacturing (casting, computer numerical control (CNC) machining, compression moulding) and additive manufacturing (SLM) including the energy consumption required for the machine’s warm-up, build and cool-down and, argon consumption required for SLM.
- Impacts of transporting the raw materials and part produced to the manufacturing locations and to the hospital.
- Indirect impacts occurred as a consequence of choosing one of the three implants such as the sterilisation process, the packaging (containing argon when needed), anesthesia required for surgery and, the instrumentation and support structure needed during the implantation (including the embodied impact of the raw materials, their processing, transportation and sterilisation).
- End-of-life impact of the implants and the instruments. Metal components are remelted and recycled to non-medical uses while the polymer parts are incinerated.

The scope excludes use phase due to the high variability of implant lifetime in both UKR and HTO interventions which depends on patient age, patient activity level, surgeon volume and expertise. There is no clear generalisable survival advantage between the two procedures. Evans et al. (2019) reports the 25-year survival rate of UKR to be 69% (95% CI 67–72) and Ollivier et al. (2021) the 20-year survival rate of HTO as ranging from 46 to 85%.

### The functional unit

The functional unit for the comparison between the AM and CM treatment options is “restoring the knee to a normal level of functional activity with an expected flexion of 120 degrees”. The procedures have the same expected outcome after surgery and overlapping lifetime indicators as seen in Section 2.1.

### Uncertainty and contribution analysis

To meet the study’s goal of comparing the expected impacts of UKRs and AM and CM HTOs in general, uncertainty about the specific value of key technical process characteristics (e.g. yields, material removal rates, electricity requirements) must be characterised, leading to uncertainty distributions for the life cycle impacts. To do this, certain foreground process characteristics are defined as uniform distributions with a lower and upper bound representing maximum and minimum values, thus describing the possible range of process inventories in a consistent way. When data is missing, proxy data is used as a central value, with a uniform distribution applied to represent the approximate nature of the proxy data. The calculations of the bounds are detailed in Sect. S1 of Online Resource 1 and can also be found in the description of processes in the foreground inventory available at: https://doi.org/10.5281/zenodo.14680968 with the file location indicated in the README file. Variability in production location and therefore the make-up of the electricity grid is modelled by scenario analysis (Section 2.6). The calculation are performed in Brightway 2 (Mutel 2017) using the background inventory of the Ecoinvent 3.8 database, allocation, cut-off by classification, version 3.8 (2021) (Wernet et al. 2016). Uncertainty in background process characteristics is taken from Ecoinvent. Results are sampled by a Monte Carlo approach (number of runs or *n* = 1000).

To assess the contribution of material, energy, transport and other factors on the total score, a function is created in Brightway 2 to perform contribution analysis as part of the Monte Carlo uncertainty analysis. The introduced method calculates the impact score of a specific subset of inputs, identified by their indices, within processes and sums their individual scores to get a cumulative total.

### Baseline inventory modelling

Data on parts, instruments and support structure types and dimensions is obtained first hand from CM UKR (Zimmer Biomet, Swindon, UK) and AM HTO (Orthoscape, Bath, UK) manufacturers. Further data on sterilisation processes and use patterns is obtained from a tertiary care reference clinical centre (Royal Devon University Healthcare NHS Foundation Trust, Exeter, UK).

Section S1 of Online Resource 1 summarises the inventory data and, where relevant, how uncertainty is modelled.

### Impact assessment

To translate emissions and resource extractions into environmental impact scores the ReCiPe 2016 version 1.03 characterisation factors are used at midpoint levels (Huijbregts et al. 2017) as implemented in ecoinvent version 3.8. This study concentrates on the climate change category characterised as global warming potential (GWP) which quantifies the integrated infrared radiative forcing increase of a greenhouse gas (GHG), expressed in kg CO$_{2e}$ (Huijbregts et al. 2017). The other 17 impact categories are included to screen for potential burden-shifting problems.

### Scenarios

In addition to the uncertainty in the data values already described, two additional scenarios are considered.

Firstly, since the carbon intensity of the electricity used during manufacturing has a significant impact on the modelled impact, a “lower-carbon” electricity scenario is modelled (termed AM $\mathrm {HTO_{g}}$, suffix *g* denoting greener electricity). Some manufacturing of these devices already occurs in Switzerland, which has a lower grid carbon intensity than the UK, so to illustrate the effect of manufacturing in different locations and grid carbonisation, in the “lower-carbon” electricity scenario, electricity inputs to the SLM and polishing processes are swapped to use Swiss Ecoinvent electricity data. This results in a significant reduction in carbon intensity. The Swiss average electricity grid mix at medium voltage has a GWP of 0.038 kg CO$_{2e}$ per kWh while the UK average electricity grid mix has a GWP of 0.307 kg CO$_{2e}$ per kWh.

Secondly, the surgical jig used with the AM HTO contributes significantly to the overall impact of the AM device. Currently, the jig is made of the same Ti alloy as the device, but in principle, this is not necessary. A scenario in which the jig is made of stainless steel is therefore also considered (termed AM HTO SJ, SJ denoting steel jig). The process electricity requirement in the case of SLM stainless steel is extracted from Baumers et al. (2010) as 31 kWh/kg of material processed. A combination of using low-carbon electricity and a stainless steel jig is also considered (AM HTO SJ$\mathrm {_{g}}$).

### Limitations of the LCA modelling

The dimensions of the CM HTO devices (plate and screws) are assumed to be the same as the AM HTO devices, as no significant differences in weight are expected between the two. The share of capital goods attributable to the interventions (related to the manufacturing of machines and facilities themselves) are not included in the analysis but their potential impact on conclusions are discussed in Sect. 4.1. Only water and argon consumption are modelled as consumables. The impact of gamma sterilisation is not included in the analysis but Leiden et al. (2020) has shown that gamma sterilisation contributes less than 1% to the impact of producing and gamma sterilising disposable medical instruments across a range of impact categories, assuming that radioactive cobalt-60 is handled disposed of safely during operation. The production of vanadium for the Ti alloy could not be modelled but vanadium only represents 4% of the alloy’s content by mass. Low-density polyethylene is used as proxy for UHMWPE as explained in Sect. S1 of Online Resource 1.

### Statistical analysis

The GWP values from the Monte Carlo modelling are examined for distribution using histograms and normality plots, showing that the data are not normally distributed. The GWP values are therefore analysed using the Krusal-Wallis test (Matlab 2022b, The MathWorks Inc., Natick, MA, USA). In the first analysis, the total GWP values for CM UKR, CM HTO and AM HTO are considered together, with post-hoc evaluation of the ranks. The total GWP values for the various scenarios are then considered i.e. CM HTO, AM HTO, AM HTO$\mathrm {_{g}}$, AM HTO SJ and AM HTO SJ$\mathrm {_{g}}$, with post-hoc evaluation of the ranks. Mann–Whitney U comparisons are made between CM HTO and AM HTO$\mathrm {_{g}}$, and between CM HTO and AM HTO SJ$\mathrm {_{g}}$. The analysis is performed for GWP values and relative GWP values considering CM HTO as the reference scenario which is set to 1.

## Results

Since our primary focus for this study is on climate change impacts, Section 3.1 first compares the results for the three devices studied. Then, Section 3.2 goes into more depth to show the impact of different manufacturing methods and scenarios. The numerical results discussed in the following sections represent median scores with a bound defined as the 25$\mathrm {^{th}}$ and 75$\mathrm {^{th}}$ interquartile range.

**Fig. 3:**
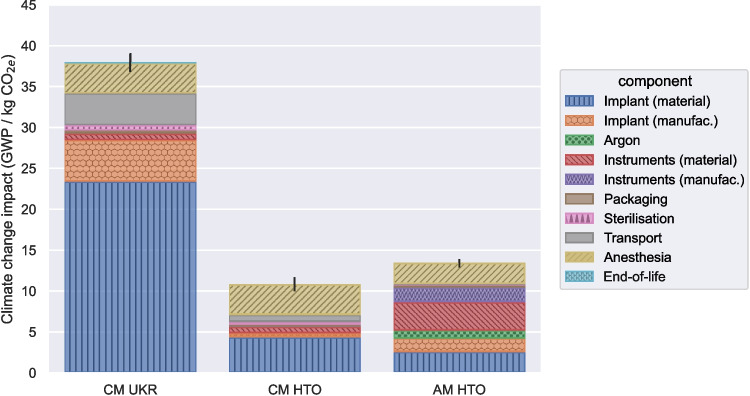
Climate change impact of CM UKR, CM HTO and AM HTO (*n* = 1000). The error bars show the 50% percentile intervals (interquartile ranges) for the total GWP. Each bar is divided into impact contributions from each component or process. Material: embodied impact of the metals and polymers procured to manufacture the implants (including scrap) shown in Fig. 1 of the UKR [1] and the HTO [2, 3] or the instruments of the CM instruments [4] and AM instruments [5]. Manufacture: impact of producing (CNC machining, SLM, casting, moulding) and post processing (coating, grinding, polishing) the implants or the instruments. Argon: impact of argon used for packaging or during SLM. Transport: impact of the transport processes shown in Fig. 2. End-of-life: End-of-life impact of recycling or incinerating implants and instruments. Instr., instruments; manufac., manufacture

**Table 1:** Mean, maximum, minimum, 25$\mathrm {^{th}}$ percentile, 75$\mathrm {^{th}}$ percentile GWP (kg CO$_{2e}$) values and interquartile ranges from Monte Carlo simulations, *n* = 1000, for the CM UKR, CM HTO and AM HTO, and three scenarios (AM HTO SJ, AM HTO$\mathrm {_{g}}$ and AM HTO SJ$\mathrm {_{g}}$)

	CM UKR	CM HTO	AM HTO	AM HTO SJ	AM HTO$\mathrm {_{g}}$	AM HTO SJ$\mathrm {_{g}}$
Median	37.93	10.68	13.37	11.94	10.48	8.33
Max	43.57	14.95	19.11	14.76	14.02	10.19
Min	32.58	8.41	11.85	10.58	9.17	7.41
25th percentile	36.88	10.00	13.01	11.66	10.18	8.12
75th percentile	38.95	11.42	13.74	12.24	10.77	8.57
IQR	2.08	1.42	0.73	0.58	0.59	0.44

*IQR* interquartile range, *SJ* steel jig, *g* greener

### Overall climate change impact of alternative devices

Figure 3 shows the range of GWP results for the three implants modelled (values given in Table 1). The Kruskal-Wallis analysis showed there are highly significant ($p<0.0001$) differences across the three main device types, the post-hoc analysis found that the differences between all devices are significant. The impacts of the CM UKR, 37.9 (36.8–38.9) kg CO$_{2e}$, are found to be highly significantly larger than the HTO devices’, CM HTO at 10.7 (10.0–11.4) kg CO$_{2e}$ and AM HTO’s at 13.4 (13.0–13.7) kg CO$_{2e}$.

As shown in Fig. 3, the impact of the three devices is dominated by material and manufacture. 29.3 (28.3–30.2) (CM UKR), 5.5 (4.9–6.3) (CM HTO) and 9.4 (9.1–9.7) (AM HTO) kg CO$_{2e}$ are attributed to the material and manufacturing impact of the implants and instruments. The GWP associated with the materials used to make the devices depends on two main factors: quantity and embodied GWP. The embodied GWP of the CoCr ingot used for the CM UKR’s femoral and tibial components is at 32.5 (31.4–33.7) kg CO$_{2e}$ per kilogram, lower than the embodied GWP of the Ti–6Al–4V powder and workpiece used for the AM HTO devices (59.4 (56.9–62.0) kg CO$_{2e}$ per kilogram) and CM HTO devices (51.8 (49.8–53.9) kg CO$_{2e}$ per kilogram). But the CM UKR implant (femoral, tibial and bearing) weighs 9.4 times more than the HTOs (plate and screws), confirming that component mass dominates the difference in GWP between CM UKR and HTO devices. The proxy used to model the UHMWPE bearing of the UKR, defined as 1–2 kg of LDPE per UHMWPE, has negligible impact on UKR results of 0.20 (0.17–0.24) kg CO$_{2e}$.

**Fig. 4:**
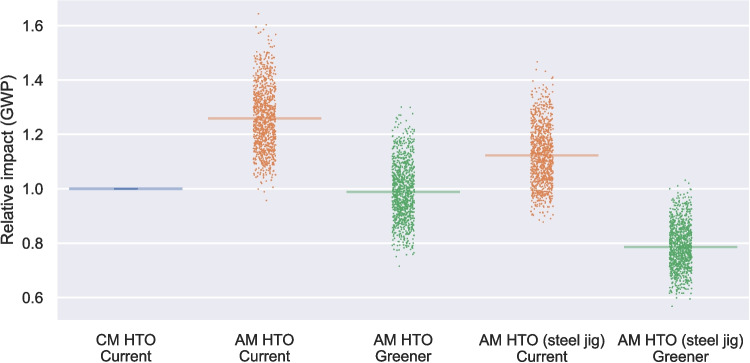
Relative GWP impacts of AM HTO relative to CM HTO impacts, in baseline, low-carbon electricity, and alternative jig material scenarios. For each run, the relative GWP impact to CM HTO is calculated (*n* = 1000) and represented as a jitter plot with the horizontal bar indicating the median

Although the estimated impact for the AM HTO is slightly higher than the CM HTO, the uncertainty ranges are substantial, and the overlap between the CM and AM HTO means that a general conclusion about the relative impact of AM cannot be drawn without further comparative analysis. Since some of the uncertainties in the GWP impact affect both the CM and AM devices simultaneously, differences are shown more clearly by plotting relative impacts for the CM and AM HTO devices, with the CM HTO having a score of 1 (Fig. 4). The first two results in Fig. 4 represent the same data as Fig. 3, but shows that the AM HTO impacts are generally slightly higher than the CM HTO ($p<0.0001$). The CM UKR relative score is 3.56 (3.33–3.79) and AM HTO is 1.25 (1.17–1.34) (Table 2).

**Table 2:** Mean, maximum, minimum, 25th percentile, 75th percentile relative values and interquartile ranges, with CM HTO as reference value (1.00), from Monte Carlo simulations, *n* = 1000, for the CM UKR, CM HTO and AM HTO, and three scenarios (AM HTO SJ, AM HTO$\mathrm {_{g}}$ and AM HTO SJ$\mathrm {_{g}}$)

	CM UKR	CM HTO	AM HTO	AM HTO SJ	AM HTO$\mathrm {_{g}}$	AM HTO SJ$\mathrm {_{g}}$
Median	3.56	1.00	1.25	1.12	0.98	0.78
Max	4.51	1.00	1.64	1.47	1.30	1.03
Min	2.74	1.00	0.96	0.88	0.72	0.57
25th percentile	3.33	1.00	1.17	1.04	0.92	0.73
75th percentile	3.79	1.00	1.34	1.19	1.05	0.84
IQR	0.47	0.00	0.17	0.15	0.14	0.11

*IQR* interquartile range, *SJ* steel jig, *g* greener

The main differences between the CM and AM HTOs visible in Fig. 3 are that, while the GWP attributed to material use is reduced, the manufacture GWP of the AM processes themselves is much higher than CM processes. The instrument (surgical jig) in the AM’s case has a much higher material impact visible in Fig. 3. There is a reduced impact from anesthetics due to the potential for faster surgery times with the customised AM HTO. These differences are explored further in the scenarios presented next.

### AM HTO contributions and scenarios

The results in Fig. 3 reflect the estimated current impact of the AM HTO, but the flexibility of the technology and wider changes in energy supply means future changes may be anticipated. Therefore, the remaining results in Fig. 4 show the reduced impacts from an AM HTO manufactured using lower-carbon electricity, using stainless steel rather than titanium alloy for the jig, and a combination of both.

#### Lower-carbon electricity scenario

There is a balance between the high operational energy requirement of the SLM process used for the AM HTO and the high scrap rate of the CM processes. The location of manufacturing makes a significant difference, given the varied and changing carbon intensities of electricity in different countries’ grids.

In the baseline scenario, the AM HTO is assumed to be produced in the UK, while the CM device is manufactured in Switzerland. Thus, while the AM process does consume about 25% more electricity than the CM route, the difference in GWP of the CM and AM devices in Fig. 3 is exaggerated, since the Swiss grid mix relies on nuclear and hydroelectric generation giving it a 66% lower impact per kWh than the UK electricity modelled.

As a comparison, the “Greener” scenario in Fig. 4 shows how the impacts of the AM device would reduce if all energy used is at the lower-carbon intensity of the Swiss mix. With current trends of reducing grid intensity, further reductions in impact would be possible from an even lower-carbon electricity supply. The Kruskal-Wallis results showed that there are highly significant ($p<0.0001$) differences in GWP values across all compared scenarios. The post-hoc analysis showed that the ranks are significantly different between all five scenarios. The Mann–Whitney U analysis showed that the median GWP for AM HTO$\mathrm {{g}}$ (10.5 (10.2–10.7) kg CO2*e*) is highly significantly ($p<0.0001$) lower than that for CM HTO (10.7 (10.0–11.4) kg CO2*e*). The effect of green energy is clearly shown by the median relative score of 0.98 (0.92–1.05) for AM HTO$\mathrm {{g}}$ compared to the reference value of 1.00 for CM HTO.

#### Stainless steel surgical guide (jig) for AM device

Figure 3 shows that instruments make a large contribution to the AM HTO’s impacts. The AM jig is a custom part, so the full burden of its production is allocated to each implantation, whereas the CM HTO instruments can be reused many times (typically 50 to 200 times). The AM jig is assumed to be printed using the same SLM machine and Ti-6Al-4V alloy as the implant, but in principle, a lower-impact material could be used. For example, stainless steel has a GWP nine times lower than the Ti-6Al-4V alloy. This scenario is shown in Fig. 4 as the “steel jig” scenario. When combined with use of lower-carbon electricity, the environmental impact (median GWP for AM HTO SJ$\mathrm {{g}}$ is 8.3 (8.1–8.5) kg CO2*e*) is highly significantly (Mann–Whitney U, $p < 0.0001$) reduced compared to CM HTO (median GWP of 10.7 (10.0–11.4) kg CO$_{2e}$). Again, the relative GWP (Table 2) shows the effect of material and greener energy, with a median relative GWP of 0.78 (0.73–0.84) compared to 1.00 for CM HTO.

#### Indirect impacts

A significant fraction of the impact of all devices is linked to the use of anesthetics during surgery. The use of the AM plate reduces surgery time by 30%, and therefore reduces the quantity of anesthetic gas needed (which has a high global warming potential of 196 kg CO$_{2e}$/l for sevoflurane) and the use of anesthetic machines, lowering the GWP of anesthesia from 3.7 (3.7–3.7) kg CO$_{2e}$ to 2.6 (2.6–2.6) kg CO$_{2e}$.

Sterilisation has a smaller impact overall, but the AM implant also enables improvements there, as only one tray is taken up by the implants in the autoclave and no other sterilisation is needed, compared to the CM instruments which take up three times more trays.

Finally, the CM HTO is manufactured partly in Switzerland and polished and packaged in the USA before being brought to the UK, so it bears significant impacts from transport by airfreight. Different manufacturing setups would have a strong influence on this impact source.

### Other impact categories

Figure S1 in online resource 1 presents estimated results for the CM and baseline AM HTO in other LCA impact categories. Generally, the trends are similar to the climate change impacts shown in more detail above, with the baseline AM HTO having similar or higher impacts than the CM HTO. Differences are due to the make-up of the electricity grid, the type or the quantity of material consumed. The 19% share of nuclear power in the UK’s electricity grid results in twice the amount of kBq of Co-60 equivalent being released into the air for the AM HTO than the CM HTO. Similarly, impact on land use for AM HTO is driven by the 6% share of biomass energy in the UK’s electricity grid. The stainless steel instruments of CM HTO doubles the impact of the CM HTO compared to the AM HTO in the terrestrial ecotoxicitiy category because of the heavy metals released to the air and water during ferronickel smelting. The AM HTO, which consumes higher quantities of Ti-6Al-4V, leads to higher impacts in other categories due to increased material use.

## Discussion

In this section, the technical, clinical, financial and policy implications of procedures for knee osteoarthritis along with environmental considerations are discussed.

### HTO in general has highly significantly lower impact than UKR

From an environmental point of view, this study has shown that HTOs yield highly significantly lower GHG emissions (maximum in this study for the AM HTO of 13.4 (13.0–13.7) kg CO$_{2e}$) than CM UKR due to a combined effect of low part size and therefore material and transport emissions, shorter surgery duration, and lower manufacturing impacts — and despite the high contribution of the single-use jig for the AM HTO (Fig. 3). The total GWP of CM UKR is 37.9 (36.8–38.9) kg CO$_{2e}$ within which 29.3 (28.3–30.2) kg CO$_{2e}$ is attributed to the material and manufacture of the implant and instruments.

Few studies have examined the environmental impact of treatments for knee osteoarthritis, with only one focusing on the implant of UKR. Lyons et al. (2021) reports emissions of 46.9 kg CO2*e* and 14.9 kg CO2*e* for the material and manufacturing of CM UKR and AM UKR implant, respectively, with the lower yield from CNC machining explaining the difference. Variations between findings can be attributed to factors such as differences in manufacturing yields and methods, the embodied impact of procured materials, and energy sources used. McGain et al. (2024) and Rizan et al. (2023) report emission of 19 kg CO$_{2e}$ and 22.7 kg CO$_{2e}$ associated with the implant of a total knee replacement, weighting around two times more than a UKR, but these studies only include the impact of the material embedded into the final part and excludes the impact of scrap generated during manufacturing. Reusable instruments are not a main contributor, with only 0.76 (0.53–1.03) kg CO$_{2e}$ emitted for a 50–200 times reuse. McGain et al. (2024) reports an emission of 1 kg CO$_{2e}$ associated with instruments reused 300 times which is aligned with this study’s finding.

For the purposes of this study, generic single-use consumables (e.g., syringes, polypropylene surgical wraps, bone cement) were excluded from the analysis as the quantity of generic single-use consumables used in HTO does not vary based on the manufacturing method (AM or CM), so it does not affect the comparison between AM and CM HTOs. Nevertheless, this factor may further amplify the difference in GHG emissions between UKR and HTO, UKR being more invasive than HTO with high revision rates as discussed in Section 4.4. These were shown to be important contributors in invasive surgeries requiring extensive post-surgery treatment, as for TKR, emitting 43.4 kg CO2*e* (Rizan et al. 2023), 34.2 kg CO2*e* (McGain et al. 2024), and 54 kg CO$_{2e}$ (Delaie et al. 2023).

### Lower-carbon impacts are possible with AM HTOs, but not guaranteed

The results show that reduced carbon emissions are not automatically delivered by switching from CM to AM. Whether AM or CM yields higher impacts depends on specific process attributes. The results specifically show that ensuring low-carbon electricity is used is important. Geographical variations in electricity grids balance results in favour of one or the other manufacturing method for GWP as shown in Section 3.2.1 but also for other impact categories as seen in Section 3.3. Concerning GWP, this is firstly because SLM has a higher operational energy requirement compared to CM, but also since in the AM approach the jig and instruments are custom and single use, making the material and manufacturing of the jig and instruments take on a new significance. There is a relatively rapid energy transition taking place in the UK. In 2022, 41% of UK electricity came from renewables compared to 2.8% in 2000 (DESNZ 2023). Thus, there is considerable potential for AM medical devices to lower environmental impact of treatment in the next few years.

The inclusion of capital good impacts in the study (CNC milling tools, printer) would not definitely shift the balance to the side of AM. To determine the share of the machine’s manufacturing, transportation and end-of-life impact that is attributable to the part, information is needed on the number of tasks performed during the machine’s lifetime and the machine’s utilisation pattern. In this study, it is assumed that the machines are powered off when not utilised (no idle electricity consumption). However, if a CNC mill or AM printer is left on idle and is under-utilised this can increase electricity consumption and the impact of the capital good attributable to the job by an order of magnitude compared to a full utilisation scenario for both the SLM and CNC mill (Faludi et al. 2015, 2017).

### AM optimisation and alternative materials offer further potential savings

The current AM design has not been fully optimised to tune it based on stress and load requirements, reduce material consumption, reduce support structure and consider post machining. Significant challenges remain in the growing domain of design for additive manufacture (DfAM) where solid modelling CAD tools are still prevalent along with the use of design methods such as design for manufacture and assembly (DfMA) that are not suitable for AM. This method, pioneered by Boothroyd and Dewhurst (Boothroyd et al. 2002) in the 1980s, radically changed how components were designed for manufacture and provided comprehensive guidelines for processes including casting, forging and machining. However, this design method has limited impact on AM design as the AM process is far more complex, the design space is considerably larger and requires a fundamental change in how components are designed (Gibson et al. 2021). This has led to the emergence of DfAM which describes new design approaches that take account of the AM process. For example, overhangs, part nesting, topology optimisation (TO), generative design and more recently, implicit modelling (Gibson et al. 2021). This approach needs to work in conjunction with understanding the commitment (resource and time) required vs the potential benefits of using AM. Designing AM components that are a direct replacement for a traditionally manufactured component will not reap the full benefits of AM but will reduce the overall commitment. A component level re-design begins to consider aspects such as light-weighting through lattice integration and topology optimisation. The commitment in terms of design expertise begins to increase but so do the potential benefits. A complete systems-level re-design goes even further by optimising the design in terms of performance, dematerialisation (material reduction), assembly, accessibility and manufacturability both from the perspective of AM and post processing. The commitment here is considerable and requires new DfAM focused design tools and expertise to maximise the full potential of AM.

At a component or systems-level re-design, and focusing on HTO plates and the associated instrumentation and jigs as detailed in this paper, a range of different AM methods and approaches can be explored. Segmenting this into different design methods, tools and skill-set allows identifying where the major gains can be realised. To enable significant light-weighting and performance optimisation (stress and load mapping) TO and lattice integration can be used. With TO the design space can be set up to ensure that material is only placed where it is required while adhering to rules that govern where high levels of stress and fatigue are likely to occur on the component. Lattice integration through implicit modelling, which represents geometry through mathematical functions and defines the relationship between points in space using constraints and conditions (Altair 2024), can be used in both a light-weighting and performance improvement capacity. For example, enabling functional grading, where a lattice density map is adjusted to ensure strength where required and incorporation of auxetic structures that enable additional geometrical performance and enhancements in the form of negative Poisson’s ratios. This structural phenomenon could be used to enhance the behaviour of the HTO when in use, e.g., creating an embedded structure that can counteract certain compressive and tensile forces. Both the instrumentation and jigs can benefit from TO and lattice integration, but challenges remain in the use of software to do this effectively. Nonetheless, if these could be implemented successfully benefits would be further reduced material mass and hence environmental impacts.

There are also financial challenges in transitioning to AM technologies in resource-constrained settings which include primary costs such as the printer and post processing requirements and, also, secondary aspects including up-skilling of the workforce and dedicated infrastructure. But it is manufacturers producing high value personalised products at low volumes that can substantially benefit from AM (Tofail et al. 2018) and can achieve cost-effectiveness by optimising build volume configurations to ensure efficient use of capacity (Ding et al. 2021).

The material used for the jig cannot come from recyclable sources, are single use and can be energy-intensive to manufacture. Ti-6Al-4V alloy is used for practical (easier to print when compared with other materials and easily accessible) and established biocompatibility reasons (Zhong et al., 2024), even though other materials could work just as well. Here stainless steel, an established material for bio-compatibility with a 9x lower impacts then Ti-6Al-4V, was analysed as an alternative. There are examples of surgical guides manufactured using high heat resistant and sterilisable resins such as acrylonitrile butadiene styrene (ABS) resin potentially further decreasing GWP due to lower embodied carbon and manufacturing energy intensity. However, ABS resin would have to go through a long period of approval for bio-compatibility and lack the strength and stiffness required to transmit load, withstand drilling and cutting and, ensure proper alignment. Additionally, end-of-life recycling is less widespread for ABS which goes against the NHS target to reduce single-use plastic consumption.

Material selections also play a significant role in the broader manufacturing challenges and cost. Here, manufacturing challenges go beyond just the AM aspects and extend to the post processing requirements which are integral for achieving precise tolerances and surface finishes. For example, Ti-6Al-4V is a tougher material to machine (after 3D printing) due to its low thermal conductivity, which elevates cutting tool temperatures and increases cutting forces. This leads to rapid tool degradation and failure resulting in higher costs. As the force to machine Ti-6Al-4V is also typically higher, more energy is required.

### Environmental, clinical and organisational benefits appear possible

This study has focused on the environmental impacts of CM UKR, CM HTO and AM HTO; however, it is important to point out other impacts from the choice of treatment for knee OA. As stated previously, knee OA is responsible for a large personal and social burden, and is a leading cause of disability and is associated with excess mortality (Palazzo et al. 2016; GBD 2021 Osteoarthritis Collaborators 2023). Knee OA incidence is projected to dramatically increase in the next three decades (GBD 2021 Osteoarthritis Collaborators 2023). The importance of physical activity for overall health has been firmly established with a solid evidence base (Warburton et al. 2006), and treating symptomatic knee OA is an important enabler of activity. Inactivity is a leading risk factor for poor health status with multiple comorbidity, which significantly increases healthcare costs and resource utilisation (Soley-Bori et al. 2020).

Joint replacement, such as UKR, is only advised at end-stage knee OA, when there is bone–on–bone contact (Campi et al. 2018). Knee OA is a degenerative condition and it may take decades between the onset of symptoms to progress to end-stage disease, and one of the reasons for increased mortality associated with knee OA is considered to be restriction in physical activity due to pain. Whilst radiographic grading systems have been established for determining disease progression (Kellgren and Lawrence 1957), these have no correlation with symptoms and there is a recommendation for early treatment (Mahmoudian et al. 2021). HTO can be successfully performed at much earlier stages in the disease process (Zhang et al. 2023), and thus provide relief from symptoms and return to activity much sooner.

UKR is a relatively complex surgery compared to TKR, and has a 2.5 times higher revision rate than TKR (Hunt et al. 2021). As seen in Section 2.1, there is no clear generalisable survival advantage between UKR and HTO procedures. Several factors are associated with an increased risk of early revision, including surgeon volume and patient age (Evans et al. 2019). Low-volume surgeons and younger patients are associated with higher early revision risk. It is important to bear in mind that revision knee surgery is several times more costly than primary surgery and also has worse outcomes for patients.

Potentially HTO has much to offer, providing sufferers with relatively early knee OA with clinically proven relief from symptoms and return to activity, improving their overall health status. Standard generic HTO surgery is also considered to be challenging surgery: the motivation for the digitally planned personalised AM HTO is to simplify the technique and enable more surgeons to be confident in offering this treatment (Zaffagnini et al. 2022). Greater access to effective treatments at earlier stages of the disease will become increasingly important as the prevalence of knee OA increases. Zaffagnini et al. (2023) have shown that AM HTO gives excellent clinical outcomes and rapid patient recovery time, with patient-reported outcomes back to normal levels at 6 months post-surgery.

There are challenges in implementing AM HTO clinically, including that personalised HTO requires pre-operative CT scans to be taken and transferred to the manufacturer. There is potentially a higher cost, but it is worth noting that UKR costs are greater than either CM or AM HTO. These potential higher costs are offset by the greater healthcare benefits of treating patients earlier in the disease process, enabling them to remain active and avoid comorbidities due to pain-related functional restriction.

The impact of the COVID pandemic is still present, with the NHS continuing to have record waiting lists for surgery (The Lancet Rheumatology 2021), this has had considerable negative impact on the lives of patients and their future health outcomes. Approaches such as personalised AM HTO, with shorter operating times, less consumption of resources and lower GWP may be important to reduce waiting lists whilst also working towards net zero targets.

## Conclusions

This study investigates whether additive manufacturing decreases the environmental harm associated with HTO relative to another type of intervention (CM UKR) for knee osteoarthritis. Beyond the surgical devices, the effect of the manufacturing method on production location, sterilisation type, packaging, surgical instrument requirement and surgery length is included. The study examines whether a clear conclusion can be made on the benefit of using AM or CM, whether the outcome is contingent on specific choices. For this, inventories are defined with uncertainty and scenarios covering process characteristics and materials, integrated with data from manufacturers and a hospital.

The study concludes that the CM UKR yields higher climate change impact than the CM HTO and the AM HTO. Choosing AM does not automatically mean lower climate change impacts for HTOs and this trend is observed for other impacts categories. AM HTO has a 1.25 (1.17–1.34) times higher GWP than CM HTO in the baseline scenario. This is partly due to the personalised single-use jig of the AM HTO, compared to the reusable instruments of the CM HTO, and the geographical location of manufacturing. Manufacturing the AM HTO with the same low-carbon Swiss electricity grid than the CM HTO brings AM HTO to overlap with the GWP of CM HTO. Changing both the electricity mix and the material used in the manufacturing of the personalised surgical jig is shown to have the potential to reduce the GWP of AM HTO to 0.78 (0.73–0.84) of that of CM HTO.

The environmental benefit of optimising the personalised AM HTO plate and jig to reduce metal consumption is highlighted. One straightforward way is to switch Ti-6Al-4V to a lower-carbon material. But to maximise the full potential of AM, component and system-level re-design have to be considered with a ’clean sheet’ design philosophy, considering design approaches such as generative design, lattice design and topology optimisation. These design methods would enable significant lightweighting through material reduction but, to use these methods effectively, new types of design skill-set are required which are currently in limited supply.

Surgical decision-making requires consideration of many factors, amongst which the likiehood of good outcomes for the patient is key. Given that people suffering with knee OA will increase, earlier treatment with AM HTO has considerable potential for relieving symptoms and restoring function as well as lowering the GWP of treatment. Current guidelines consider joint replacement, in particular TKR, as effective treatments for knee OA, but overlook the impact of delaying treatment until end-stage before joint replacement is recommended. Future guidelines will need to embrace environmental consequences.

To provide data to inform policy changes, future research should combine health economics studies investigating efficacy of early stage treatment of knee OA using HTO and LCA studies including the consequences of untreated symptomatic knee OA as well as the long-term effects of treatment with HTO.

## Supplementary information

Additional supplementary information is linked to the article on the journal’s website. Online resource 1 provides information on the baseline inventory and normalised results in the 18 LCA impact categories for the CM HTO and baseline AM HTO.

## Supplementary Information

Below is the link to the electronic supplementary material.

Supplementary file 1 (pdf 1053 KB)

## Data Availability

Primary foreground inventory data obtained first hand from manufacturers and a hospital are treated as confidential. Secondary foreground inventory data obtained from literature that support the findings of this study are openly available in LCA-knee-OA-treatment at https://doi.org/10.5281/zenodo.14680968 along with calculations. Background inventory data from Ecoinvent version 3.8 are referenced in the repository but were used under a license and so are not publicly available.

## Abbreviation

OA: Osteoarthritis
UKR: Unicompartmental knee replacement
CM: Conventionally manufactured
HTO: High tibial osteotomy
TKR: Total knee replacement
AM: Additive manufactured
CM: Conventionally manufactured
NHS: National Health Service
LCA: Life cycle assessment
CNC: Computer numerical control
SLM: Selective laser melting
CoCr: Cobalt chrome
UHMWPE: Ultra high molecular weight polyethylene
g: Greener
SJ: Stainless steel jig
ABS: Acrylonitrile butadiene styrene
DfAM: Design for additive manufacture
DfMA: Design for manufacture and assembly
TO: Topology optimisation
GWP: Global warming potential
LDPE: Low-density polyethylene
GHG: Greenhouse gas
IQR: Interquartile range

## References

[CR1] Altair (2024) What is implicit modelling? https://help.altair.com/inspire/en_us/topics/implicit/implicit_modeling_r.htm

[CR2] Baumers M, Tuck CJ, Hague RJM, Ashcroft I, Wildman R (2010) A comparative study of metallic additive manufacturing powerconsumption. In: Proc of the solid freeform fabr symp, pp 278–288. Austin, TX

[CR3] Baumers M, Tuck C, Wildman R, Ashcroft I, Hague R (2011) Energy inputs to additive manufacturing: does capacity utilization matter? 22nd Annu int solid freeform fabr symp

[CR4] Blagojevic M, Jinks C, Jeffery A, Jordan KP (2010) Risk factors for onset of osteoarthritis of the knee in older adults: a systematic review and meta-analysis. Osteoarthr Cartil 18(1):24–33. 10.1016/j.joca.2009.08.010 10.1016/j.joca.2009.08.010 19751691

[CR5] Boothroyd G, Dewhurst P, Knight W (2002) Product design for manufacture and assembly, 2nd edn. M. Dekker, New York

[CR6] Campi S, Tibrewal S, Cuthbert R, Tibrewal SB (2018) Unicompartmental knee replacement - current perspectives. J Clin Orthop Trauma 9(1):17–23. 10.1016/j.jcot.2017.11.013 29628678 10.1016/j.jcot.2017.11.013 PMC5884047

[CR7] Delaie C, Cerlier A, Argenson JN, Escudier JC, Khakha R, Flecher X, Jacquet C, Ollivier M (2023) Ecological burden of modern surgery: an analysis of total knee replacement’s life cycle. Arthroplasty Today 23:101187. 10.1016/j.artd.2023.101187 37745969 10.1016/j.artd.2023.101187 PMC10514426

[CR8] DESNZ (2023) Electricity: commodity balances (Digest of UK Energy Statistics (DUKES) 5.1). Department for energy security and net zero. https://www.gov.uk/government/statistics. Accessed on 19 Jul 2024

[CR9] Ding J, Baumers M, Clark EA, Wildman RD (2021) The economics of additive manufacturing: towards a general cost model including process failure. Int J of Prod Econ 237:108087. 10.1016/j.ijpe.2021.108087

[CR10] Evans JT, Walker RW, Eva JP, Blom AW, Sayers A, Whitehouse MR (2019) How long does a knee replacement last? A systematic review and meta-analysis of case series and national registry reports with more than 15 years of follow-up. Lancet 393(1):655–663. 10.1016/S0140-6736(18)32531-5 30782341 10.1016/S0140-6736(18)32531-5 PMC6381229

[CR11] Faludi J, Bayley C, Bhogal S, Iribarne M (2015) Comparing environmental impacts of additive manufacturing vs traditional machining via life-cycle assessment. Rapid Prototyp J 21(1):14–33

[CR12] Faludi J, Baumers M, Maskery I, Hague R (2017) Environmental impacts of selective laser melting: do printer, powder, or power dominate? J of Ind Ecol 21(S1):S144–S156. 10.1111/jiec.12528

[CR13] Ford S, Despeisse M (2016) Additive manufacturing and sustainability: an exploratory study of the advantages and challenges. J Clean Prod 137:1573–1587. 10.1016/j.jclepro.2016.04.150

[CR14] GBD 2021 Osteoarthritis Collaborators (2023) Global, regional, and national burden of osteoarthritis, 1990–2020 and projections to 2050: a systematic analysis for the global burden of disease study 2021. Lancet 5(9) 10.1016/S2665-9913(23)00163-7 PMC10477960 37675071

[CR15] Gibson I, Rosen D, Stucker B, Khorasani M (2021) Des for addit manuf, pp 555–607. Springer International Publishing

[CR16] GrandViewResearch (2022) Healthcare additive manufacturing market size, share and trends analysis report by technology, by application, by material, by region, and segment forecasts, 2022 - 2030. https://www.grandviewresearch.com/industry-analysis/healthcare-additive-manufacturing-market. Accessed on 19 Feb 2023

[CR17] Gutowski T, Jiang S, Cooper D, Corman G, Hausmann M, Manson J-A, Schudeleit T, Wegener K, Sabelle M, Ramos-Grez J, Sekulic DP (2017) Note on the rate and energy efficiency limits for additive manufacturing: rate and energy efficiency limits for am. J Ind Ecol 21(S1):S69–S79. 10.1111/jiec.12664

[CR18] Huijbregts M, Steinmann Z, Elshout P et al (2017) Recipe 2016: a harmonised life cycle impact assessment method at midpoint and endpoint level. Int J Life Cycle Ass 22(S1):138–147. 10.1007/s11367-016-1246-y

[CR19] Hunt LP, Blom AW, Matharu GS, Kunutsor SK, Beswick AD, Wilkinson JM, Whitehouse MR (2021) Patients receiving a primary unicompartmental knee replacement have a higher risk of revision but a lower risk of mortality than predicted had they received a total knee replacement: data from the national joint registry for England, Wales, Northern Ireland, and the isle of man. J Arthroplasty 36(2):471-477.e6. 10.1016/j.arth.2020.08.063 33011013 10.1016/j.arth.2020.08.063

[CR20] Ingarao G (2018) Environmental modelling of aluminium based components manufacturing routes: additive manufacturing versus machining versus forming. J Clean Prod 176:261–276. 10.1016/j.jclepro.2017.12.115

[CR21] ISO14040:2006 (2022) ISO 14040:2006 environmental management — life cycle assessment — principles and framework. https://www.iso.org/standard/37456.html. Accessed on 7 Feb 2023

[CR22] Katsuura Y, Qureshi SA (2020) Additive manufacturing for metal applications in orthopaedic surgery. J Am Acad Orthop Surg 28(8):e349–e355. 10.5435/JAAOS-D-19-00420 31939752 10.5435/JAAOS-D-19-00420

[CR23] Kellens K, Mertens R, Paraskevas D, Dewulf W, Duflou JR (2017) Environmental impact of additive manufacturing processes: does am contribute to a more sustainable way of part manufacturing? Proced CIRP 61:582–587. 10.1016/j.procir.2016.11.153

[CR24] Kellens K, Yasa E, Renaldi Dewulf, W, Kruth J, Duflou J (2011) Energy and resource efficiency of SLS/SLM processes. 22nd Annu Int Solid Freeform Fabr Symp, pp 1–16

[CR25] Kellgren JH, Lawrence JS (1957) Radiological assessment of osteo-arthrosis. Ann Rheum Dis 16(4):494–502. 10.1136/ard.16.4.494 13498604 10.1136/ard.16.4.494 PMC1006995

[CR26] Leiden A, Cerdas F, Noriega D, Beyerlein J, Herrmann C (2020) Life cycle assessment of a disposable and a reusable surgery instrument set for spinal fusion surgeries. Resour Conserv Recycl 156:104704. 10.1016/j.resconrec.2020.104704

[CR27] Lyons R, Newell A, Ghadimi P, Papakostas N (2021) Environmental impacts of conventional and additive manufacturing for the production of Ti-6Al-4V knee implant: a life cycle approach. Int J Adv Manuf Tech 112(3–4):787–801. 10.1007/s00170-020-06367-7

[CR28] Mahmoudian A, Lohmander LS, Mobasheri A, Englund M, Luyten FP (2021) Early-stage symptomatic osteoarthritis of the knee - time for action. Nat Rev Rheumatol 17(10):621–632. 10.1038/s41584-021-00673-4 34465902 10.1038/s41584-021-00673-4

[CR29] McGain F, Wickramarachchi K, Aye L, Chan BG, Sheridan N, Tran P, McAlister S (2024) The carbon footprint of total knee replacements. Aust Health Rev. 10.1071/AH24154 10.1071/AH24154 39467327

[CR30] Meng M, Wang J, Huang H, Liu X, Zhang J, Li Z (2023) 3D printing metal implants in orthopedic surgery: methods, applications and future prospects. J Orthop Translat 42:94–112. 10.1016/j.jot.2023.08.004 37675040 10.1016/j.jot.2023.08.004 PMC10480061

[CR31] Mutel C (2017) Brightway: an open source framework for life cycle assessment. J Open Source Softw 2(12):236. 10.21105/joss.00236

[CR32] NHS (2020) Delivering a ‘net zero’ national health service. https://www.england.nhs.uk/greenernhs/publication/delivering-a-net-zero-national-health-service/. Accessed on 19 Feb 2023

[CR33] Ollivier B, Berger P, Depuydt C et al (2021) Good long-term survival and patient-reported outcomes after high tibial osteotomy for medial compartment osteoarthritis. Knee Surg Sports Traumatol Arthrosc 29:3569–3584. 10.1007/s00167-020-06262-4 32909057 10.1007/s00167-020-06262-4

[CR34] Palazzo C, Nguyen C, Lefevre-Colau M-M, Rannou F, Poiraudeau S (2016) Risk factors and burden of osteoarthritis. Ann Phys Rehab Med 59(3):134–138. 10.1016/j.rehab.2016.01.006. Special Issue: Osteoarthritis / Coordinated by Emmanuel Coudeyre and François Rannou 10.1016/j.rehab.2016.01.006 26904959

[CR35] Patel A, Pavlou G, Mújica-Mota RE, Toms AD (2015) The epidemiology of revision total knee and hip arthroplasty in England and Wales: a comparative analysis with projections for the United States. A study using the national joint registry dataset. Bone Joint J 97-B(8):1076–81. 10.1302/0301-620X.97B8.35170 10.1302/0301-620X.97B8.35170 26224824

[CR36] Priarone PC, Ingarao G, di Lorenzo R, Settineri L (2017) Influence of material-related aspects of additive and subtractive ti-6al-4v manufacturing on energy demand and carbon dioxide emissions. J Ind Ecol 21(S1):S191–S202. 10.1111/jiec.12523

[CR37] Rizan C, Lillywhite R, Reed M, Bhutta MF (2023) The carbon footprint of products used in five common surgical operations: identifying contributing products and processes. J R Soc Med 116(6):199–213. 10.1177/01410768231166135 10.1177/01410768231166135 PMC10331364 37054734

[CR38] Soley-Bori M, Ashworth M, Bisquera A, Dodhia H, Lynch R, Wang Y, Fox-Rushby J (2020) Impact of multimorbidity on healthcare costs and utilisation: a systematic review of the UK literature. Br J Gen Pract 71:e39–e46. 10.3399/bjgp20X713897 33257463 10.3399/bjgp20X713897 PMC7716874

[CR39] The Lancet Rheumatology (2021) Too long to wait: the impact of COVID-19 on elective surgery. Lancet Rheumatol 3(2):e83. 10.1016/S2665-9913(21)00001-1 33778775 10.1016/S2665-9913(21)00001-1 PMC7987531

[CR40] Tofail SA, Koumoulos EP, Bandyopadhyay A, Bose S, O’Donoghue L, Charitidis C (2018) Additive manufacturing: scientific and technological challenges, market uptake and opportunities. Mater Today 21(1):22–37. 10.1016/j.mattod.2017.07.001

[CR41] Van Sice C, Faludi J (2021) Comparing environmental impacts of metal additive manufacturing to conventional manufacturing. Proc Des Soc 1:671–680. 10.1017/pds.2021.67

[CR42] Warburton DE, Nicol CW, Bredin SS (2006) Health benefits of physical activity: the evidence. CMAJ 174(6):801–9. 10.1503/cmaj.051351 16534088 10.1503/cmaj.051351 PMC1402378

[CR43] Wernet G, Bauer C, Steubing B, Reinhard J, Moreno-Ruiz E, Weidema B (2016) The ecoinvent database version 3 (part I): overview and methodology. Int J Life Cycle Assess 21(9):1218–1230. 10.1007/s11367-016-1087-8

[CR44] White SH, Ludkowski PF, Goodfellow JW (1991) Anteromedial osteoarthritis of the knee. J Bone Joint Surg Br 73(4):582–6. 10.1302/0301-620X.73B4.2071640 2071640 10.1302/0301-620X.73B4.2071640

[CR45] Zaffagnini S, Dal Fabbro G, Lucidi GA, Agostinone P, Belvedere C, Leardini A, Grassi A (2023) Personalised opening wedge high tibial osteotomy with patient-specific plates and instrumentation accurately controls coronal correction and posterior slope: results from a prospective first case series. Knee 44:89–99. 10.1016/j.knee.2023.07.011 37562120 10.1016/j.knee.2023.07.011

[CR46] Zaffagnini S, Dal Fabbro G, Belvedere C, Leardini A, Caravelli S, Lucidi GA, Agostinone P, Mosca M, Neri MP, Grassi A (2022) Custom-made devices represent a promising tool to increase correction accuracy of high tibial osteotomy: a systematic review of the literature and presentation of pilot cases with a new 3D-printed system. J Clin Med 11(19). 10.3390/jcm11195717 10.3390/jcm11195717 PMC9571741 36233583

[CR47] Zhang B, Qian H, Wu H, Yang X (2023) Unicompartmental knee arthroplasty versus high tibial osteotomy for medial knee osteoarthritis: a systematic review and meta-analysis. J Orthop Surg 31(1). 10.1177/10225536231162829 10.1177/10225536231162829 36893443

[CR48] Zimmer Biomet (2024) Zimmer patient specific intruments. https://www.hungerfordmd.com/zimmer-instruments.html. Accessed on 24 Jul 2024

