# Supplementary material for: High tibial osteotomy and additive manufacture can significantly reduce the climate impact of surgically treating knee osteoarthritis
Source: Int J Life Cycle Assess. 2025 Jun 13;30(7):1651–65. doi: 10.1007/s11367-025-02473-4 (PMC12274144; doi:10.1007/s11367-025-02473-4)
Supplement: Supplementary file 1 — (pdf 1053 KB) [file 11367_2025_2473_MOESM1_ESM.pdf]

Supplementary information for "High tibial osteotomy and additive manufacture can significantly reduce the climate impact of surgically treating knee osteoarthritis"

R. L. Anspach[1, 2,*], H. S. Gill[1, 3, 4], V. Dhokia[1, 5], and R. C. Lupton[1, 2]

[1]Department of Mechanical Engineering, University of Bath, Claverton Down, Bath, BA2 7AY, UK

[2]Institute of Sustainability and Climate Change, University of Bath, Claverton Down, Bath, BA2 7AY, UK

[3]Centre for Therapeutic Innovation, University of Bath, Claverton Down, Bath, BA2 7AY, UK

[4]Centre for Bioengineering & Biomedical Technologies, University of Bath, Claverton Down, Bath, BA2 7AY, UK

[5]Centre for Digital, Manufacturing & Design, University of Bath, Claverton Down, Bath, BA2 7AY, UK

* email: rla44@bath.ac.uk

This supporting information summarises the inventory data and, where relevant, how uncertainty is modelled. It also provides normalised results in the 18 midpoint impact categories for the CM HTO and baseline AM HTO (where for each category the total impact of the CM HTO is normalised to 1).

S.1 Baseline inventory

S.1.0.1 Manufacturing locations and transport

Medical grade metal alloy ingots such as Ti-6Al-4V and cobalt chrome (CoCr) are modelled to be produced and, when necessary, forged into a workpiece in Russia where data is most established. However, it should be noted that geopolitical tensions are causing a shift in the market to other producing countries. The production location of cold-rolled stainless steel, ultra high molecular weight polyethylene (UHMWPE), CoCr casting, and UHMWPE compression moulding are less certain. They are modelled as being produced

in Europe and transported from and to Central Europe. The metal alloys are transported to Wales for gas atomisation and SLM, and to Switzerland for CNC machining, using 16–32 metric ton lorries. Once cast, moulded or machined CM devices and instruments are flown by airfreight to the North-Eastern coast of the United-States (US) for grinding, polishing, plasma coating, gamma sterilisation and packaging while AM devices including instruments and guide (jig) are entirely produced, packed and sterilised in the UK. Implantation and autoclave sterilisation for the devices that are not gamma sterilised take place in UK hospitals. The inventories for transport by lorry in continental Europe and global airfreight are taken from Ecoinvent (Wernet et al., 2016).

S.1.0.2 Electricity and heat generation

All manufacturing processes are modeled to consume regional (US-Northeast Power Coordinating Council), country (Swiss, Russian and British) or European average electricity and heat mixes.

S.1.0.3 Raw material production

The mining and production of pure metals are taken from the Ecoivent 3.8 database (Wernet et al., 2016). The metal alloying processes, synthetic polymer, workpiece and powder production processes are modelled with the following data:

- **Ti-6Al-4V ingot production:** Ti-6Al-4V is a metal alloy with a high specific strength and excellent corrosion resistance that is widely employed for medical implants. Ti-6Al-4V is composed of 90% titanium, 6% aluminium, 4% vanadium and 2.5% iron (KYOCERA SGS Precision Tools Europe, 2023). To produce Ti-6Al-4V, titanium is extracted from titanium tetrachloride and high-grade titanium slag by the Kroll process. Then, Ti-6Al-4V alloy is obtained by vacuum arc remelting the pure metals (Muller, 2008). The alloy is then moulded and cooled down naturally to obtain an ingot.
- **CoCr ingot production:** The cobalt-chrome-molybdenum alloy is composed of 28% chromium, 60% cobalt and 7% molybdenum (Dolgov et al., 2016). Pure metals for CoCr production are melted together in an electric arc furnace before being poured into a mould and cooled-down naturally to form an ingot. The heat and electricity requirement of the electric arc melting process is adapted from Primas (2020) to use average Russian electricity grid mix.
- **Stainless steel ingot production:** 18/8 grade stainless steel is composed of 28% ferronickel and 23% ferrochromium and 49% iron from recycled sources. These metals are loaded into an electric arc furnace and melted together before being purified in an argon-oxygen decarburisation refining vessel. The stainless steel is then shaped by cold rolling. The process of melting, purifying and cold rolling stainless steel is taken from ecoinvent (Althaus, 2023). Instruments and guide (jig) can

contain metals from recycled sources as opposed to implants where high purity has to be assured.

- **UHMWPE production:** Low-density polyethylene (LDPE) was used as proxy for ultra high molecular weight polyethylene. To account for the higher energy requirement of producing UHMWPE compared to low-density polyethylene, a range of 1–2 kg of low-density polyethylene production is assumed to be required to obtain 1 kg of UHMWPE.
- **Workpiece production:** To convert a metal ingots into a workpiece of appropriate size, a forging rolling process is commonly used which is estimated by Ashby (2013) to have a processing energy of 14.5 MJ/kg of metal processed.
- **Powder production:** For additive manufacturing Ti-6Al-4V ingot has to be reduced into powder. The reduction of Ti-6Al-4V into powder is considered with the gas atomisation route. Gas atomisation is a process where a Ti-6Al-4V ingot is rotated into an inductive coil which melts the bar. The molten metal is then atomised by high pressure argon jets which split the molten metal into fine droplets. The metal powder is then separated from the argon by cyclonic separation and sieved to filter particles with the correct size and morphology for additive manufacturing from oversize and undersized powder (Cappucci et al., 2020). The electrical energy requirement for gas atomisation has been estimated to be between 30.1 and 33.3 MJ/kg of metal processed (Granta Design, 2010).

S.1.0.4 Additive manufacturing and conventional manufacturing

Selective laser melting The additive manufactured plate, instruments and jig are printed by SLM where metal powder is melted by the energy of a laser beam. SLM starts with a building platform where thin layers of metal powder are spread with a recoater blade. The thin layers are then melted together with a high-power laser beam and the process is repeated until the layers form the required three dimensional object. The process is performed in a tightly controlled chamber containing an inert gas, usually argon, to avoid oxidation. An air filtering system is operated continuously to guarantee the right level of argon purification. Before printing, the build chamber is filled of argon to flush out any other contaminants. Argon consumption was modeled as in Faludi et al. (2017). Once the printing is performed the part is manually extracted from the chamber and loose powder is collected for future reuse. Waste powder that is caught in protective equipment or during machine cleaning is sent to recycling.

Table 1 Process electricity consumption of SLM for Ti-6Al-4V. Sources: Maskery et al. (2015) and Faludi et al. (2017)

	Low process rate	High process rate	Sources
Deposition rates – build	2.4 mm^3/s	4.5 mm^3/s	Maskery et al. (2015)
Warm-up time	30 min		
Cool-down time	240 min		
Build mean power consumption	986 W		Faludi et al. (2017)
Warm-up power consumption	738 W		
Cool-down power consumption	430 W		
Energy consumption per kg	98.14 MJ/kg	52.34 MJ/kg	Calculated

Following the approach of Faludi et al. (2017), the SLM energy requirement is modelled including three operational modes, warm-up, build and cool-down time. Firstly, material properties are important to consider when calculating the energy requirement of SLM a part. In the case of the Ti-6Al-4V parts, the electricity requirement of the SLM is calculated using a high and low value for the deposition rate, as shown in Table 1, which is a function of the material, layer thickness, hatch spacing, and scanning speed.

CNC machining CM HTO plates and screws and CM instruments are manufactured by CNC machining. CNC machining, as opposed to additive manufacturing, is a digitally controlled subtractive process. CNC machining includes milling where a spindle rotates removing material (coarse machining) and finishing processes such as grinding where an abrasive wheel removes light amounts of material. There are several methods to calculate the energy requirement of machining processes. The method of Kara and Li (2011) is used in this study which relates the specific energy consumption (SEC) to the material removal rate (MRR) according to Equation 1. MRR measures the quantity of material removed per unit of time.

$$SEC = C_0 + \frac{C_1}{\text{MRR}} \qquad [kJ/cm^3] \tag{1}$$

where C_0 and C_1 are the machine specific coefficients. The SEC is expressed as the energy consumed when the milling tool removes 1 cm^3 of material.

MRR is a function of the axial and radial depth of the cut, the cutting feed, the number of teeth, the diameter of the cutting tool and the feed per tooth. MRR is higher for coarse machining where larger pieces of material are removed and lower for fine machining. In the current study, coarse machining is modelled as being carried out using a 15 mm diameter flat end mill followed by finishing using an 8 mm diameter flat end mill (Le and Paris, 2018). The C_0 and C_1 values are extracted from Kara and Li (2011) for wet milling on the Mori Seiki Dura Vertical 5500. A low and high value is modelled for the MRR of roughing and finishing as shown in Table 2. For grinding, using a depth of 2 mm and a feed rate of 6 m/min (Guo et al., 2011), the specific grinding energy is 110 J/mm^3.

Table 2 CNC machining material removal rates and energy requirement. Sources: Kara and Li (2011), Guo et al. (2011) and Lee et al. (2013)

CNC machining		Sources
MRR roughing	0.29–0.66 cm^3/s	Kara and Li (2011)
MRR finishing	0.01–0.02 cm^3/s	Kara and Li (2011)
Rough machining energy (per unit wt removed)	6.01–9.91 kJ/cm^3	Calculated
Fine machining energy (per unit wt removed)	103.9–204.8 kJ/cm^3	Calculated
Grinding energy (per unit wt removed)	110 J/mm^3	Guo et al. (2011)
Polishing power	0.8-2.2 kW	Lee et al. (2013)

Casting The tibial and femoral component of the CM UKR implant is manufactured using investment casting (lost-wax) method which enables more precise part production compared to sand and die casting and eliminates the need for extensive machining for precision parts. Investment casting is modeled as in ecoinvent (Menard, 2021).

Compression moulding The plastic bearing of the CM UKR implant is manufactured from UHMWPE by compression moulding. The process energy requirement of compression moulding is taken from Ashby (2013) and modelled as 11–16 MJ/kg.

Plasma coating The CM UKR femoral and tibial components are plasma coated with Ti-6Al-4V. During plasma coating, a high temperature plasma jet melts and propels a powdered material onto the surface of the component creating a thin layer. Wang et al. (2020) estimates an electricity consumption of 1.71–1.81 MJ by cm^3 of surface covered.

S.1.0.5 Processing yields

Scrap production is considered at workpiece and powder production (cold rolling, gas atomisation and forging) and at the point of manufacturing during SLM, CNC machning and casting. The yield of these manufacturing processes depend on the particle size required and, the size and complexity of the shape. Gas atomisaiton is modelled with a 10–12% scrap rate. Powell et al. (2020) explains that despite only 6.7% (Sartin et al., 2017) of the powder is used after gas atomisation for AM, loose powder is recovered repeatedly until it can no longer be atomised. This results in a net scrap rate of 12.5% (Powell et al., 2020). Forging, on the other hand, does not result in any scrap produced. Kellens et al. (2011) estimates a 80% yield for SLM while for rough CNC machining variations are larger between 30–80% (Priarone et al., 2017). Investment casting results in 1/3–2/3 of the ingot into the final part (Menard, 2021). Manufacturing yields are modelled as between 76–83% for SLM, 33–66% for coarse machining, 90–95% for fine machining and 95–98% for grinding. Removal and treatment of waste and recyclable material is cut-off from the production processes, consistent with the ecoinvent cut-off by allocation system model.

S.1.0.6 Instruments reuse

CM instruments are modeled to be reused between 50 and 200 times over their lifetime.

S.1.0.7 Packaging

CM UKR and CM HTO devices are packed in an inert gas (argon) using a single-chamber vacuum bag packer modeled with a power consumption of 1–2 kW and packing 10–20 devices per minute. AM devices are packed using a manual sealing machine. The total weight and type of packaging are obtained from the CM UKR manufacturer.

S.1.0.8 Sterilisation

Surgical instruments for all types of surgeries and the AM HTO devices are generally sterilised in an autoclave at the hospital they will be used in, while the CM HTO and CM UKR devices are pre-packed and sterilised using gamma irradiation before being transported to the hospital. The power rating, cycle time and capacity of the autoclave was communicated by the hospital. The AM HTO plate, instruments and jig are modeled to only take up one tray per run time, whereas the CM instruments take up three to four trays.

S.1.0.9 Anesthesia

The implantation of the devices is carried out with general anesthesia. The emissions related to anesthetic gases, the duration and power rating of the anesthetic machine is summarised in Table 3. Sevofurlane, an anesthetic gas, is 130 times higher global warming potential (GWP) than carbon dioxide (McGain et al., 2021). The AM HTO enables to reduce the implantation time by 30% (Belvedere et al., 2023) and therefore the amount of anesthetic gas required. The implantation of the CM HTO and CM UKR is modelled to last 1.5 hours while the implantation of the AM HTO is modeled to take an hour.

Table 3 Anesthesia specifications (McGain et al., 2021)

Anesthesia	General	
Anesthesia duration	161	min
Mean di-oxygen use	197	l
Mean compressed air use	80	l
Mean sevoflurane use	24	ml liquid
Anesthetic machine	80	W
Patient air warmer	800	W
Anesthetic scavenging	400	W
Di-oxygen supply GHG emissions	0.0021	kg CO_{2e}/l
Sevoflurane GHG emissions	0.196	kg CO_{2e}/ml liquid
Compressed air supply GHG emissions	0.00051	kg CO_{2e}/l

S.2 Results for other midpoint categories

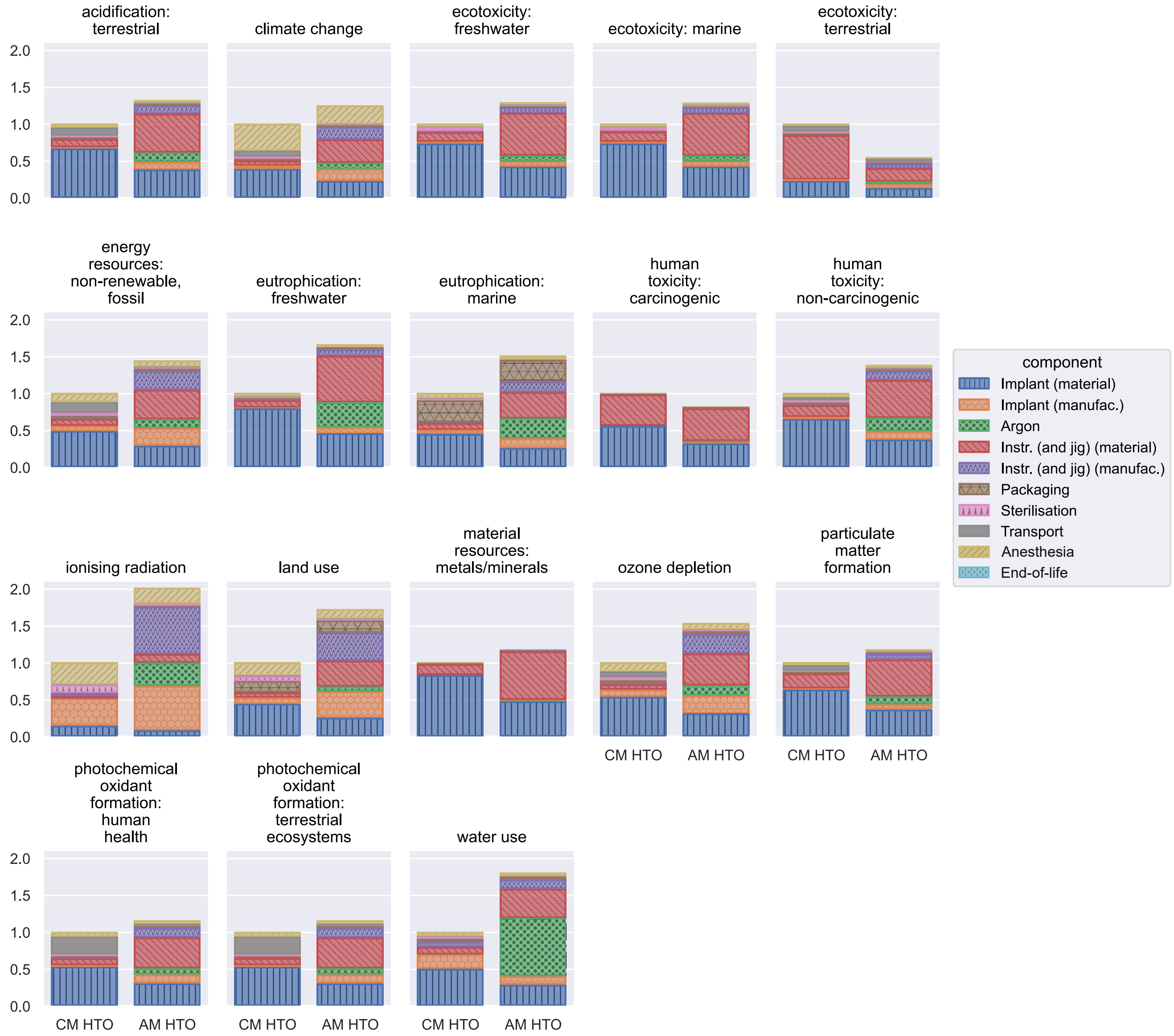

Fig. S 1 Normalised scores in 18 ReCiPe midpoint H impact categories for an AM and CM HTO. For each category the total impact of the CM HTO is normalised to 1. Instr = instruments; manufac = manufacture

References

Althaus, H.-J. (2023). Sheet rolling, chromium steel, RER, Ecoinvent version 3.8.

Ashby, M. F. (2013). Chapter 6 - Eco-data: Values, sources, precision. In Ashby, M. F., editor, *Mater and the Env*, pages 119–174. Butterworth-Heinemann, Boston, second edition.

Belvedere, C., MacLeod, A., Leardini, A., Grassi, A., Fabbro, G. D., Zaffagnini, S., and Gill, H. S. (2023). 3D medical imaging analysis, patient-specific instrumentation and individualized implant design, with additive manufacturing creates a new personalized high tibial osteotomy treatment option. *J of Mech in Med and Bio*, 0(0):2340041. `https://doi.org/10.1142/S0219519423400419`.

Cappucci, G. M., Pini, M., Neri, P., Marassi, M., Bassoli, E., and Ferrari, A. M. (2020). Environmental

sustainability of orthopedic devices produced with powder bed fusion. *J of Ind Ecol*, 24(3):681–694. https://doi.org/10.1111/jiec.12968.

Dolgov, N., Dikova, T., Dzhendov, D., Pavlova, D., and Simov, M. (2016). Mechanical properties of dental Co-Cr alloys fabricated via casting and selective laser melting. *Int Sci-Tech Conf "Innov in Eng"*, pages 3–7.

Faludi, J., Baumers, M., Maskery, I., and Hague, R. (2017). Environmental impacts of selective laser melting: Do printer, powder, or power dominate? *J of Ind Ecol*, 21(S1):S144–S156. https://doi.org/10.1111/jiec.12528.

Granta Design (2010). CES edupack version 6.2.0, 2010.

Guo, G., Liu, Z., An, Q., and Chen, M. (2011). Experimental investigation on conventional grinding of TI–6AL–4V using SiC abrasive. *Int J of Adv Manuf Tech*, 57(1-4):135–142. https://doi.org/10.1007/s00170-011-3272-z.

Kara, S. and Li, W. (2011). Unit process energy consumption models for material removal processes. *CIRP Ann*, 60(1):37–40. https://doi.org/10.1016/j.cirp.2011.03.018.

Kellens, K., Yasa, E., Renaldi, Dewulf†, W., Kruth, J., and Duflou, J. (2011). Energy and resource efficiency of sls/slm processes. *22nd Annu Int Solid Freeform Fabr Symp*, pages 1–16.

KYOCERA SGS Precision Tools Europe (2023). Ti–6Al–4V (grade 5) titanium alloy data sheet. https://kyocera-sgstool.co.uk/titanium-resources/titanium-information-everything-you-need-to-know/ti-6al-4v-grade-5-titanium-alloy-data-sheet/. Accessed on 6 July 2023.

Le, V. T. and Paris, H. (2018). A life cycle assessment-based approach for evaluating the influence of total build height and batch size on the environmental performance of electron beam melting. *Int J of Adv Manuf Tech*, 98(1-4):275–288. https://doi.org/10.1007/s00170-018-2264-7.

Lee, H., Park, S., and Jeong, H. (2013). Evaluation of environmental impacts during chemical mechanical polishing (cmp) for sustainable manufacturing. *J Mech Sci Technol*, 27(S1):511–518. https://doi.org/10.1007/s12206-012-1241-6.

Maskery, I., Aremu, A., Simonelli, M., Tuck, C., Wildman, R., Ashcroft, I., and Hague, R. (2015). Mechanical properties of TI–6AL–4V selectively laser melted parts with body-centred-cubic lattices of varying cell size. *Exp Mech*, 55. https://doi.org/10.1007/s11340-015-0021-5.

McGain, F., Sheridan, N., Wickramarachchi, K., Yates, S., Chan, B., and McAlister, S. (2021). Carbon Footprint of General, Regional, and Combined Anesthesia for Total Knee Replacements. *Anesthesiol*, 135(6):976–991. https://doi.org/10.1097/ALN.0000000000003967.

Menard, J.-F. (2021). casting, steel, lost-wax, RoW, Ecoinvent version 3.8.

Muller, F Weingarnter, E. (2008). Vacuum arc melting and remelting process. ASM international. pages 132 – 138.

Powell, D., Rennie, A. E., Geekie, L., and Burns, N. (2020). Understanding powder degradation in metal additive manufacturing to allow the upcycling of recycled powders. *J of Clean Prod*, 268:122077. https://doi.org/10.1016/j.jclepro.2020.122077.

Priarone, P. C., Ingarao, G., di Lorenzo, R., and Settineri, L. (2017). Influence of material-related aspects of additive and subtractive ti-6al-4v manufacturing on energy demand and carbon dioxide emissions. *J of Ind Ecol*, 21(S1):S191–S202. https://doi.org/10.1111/jiec.12523.

Primas, A. (2020). Iron-nickel-chromium alloy production, RoW, Ecoinvent version 3.8. Accessed on 7 February 2023.

Sartin, B., Pond, T., Griffith, B., Everhart, W., Elder, L., Wenski, E., Cook, C., Wieliczka, D., King, W., Rubenchik, A., et al. (2017). 316l powder reuse for metal additive manufacturing. *Int Solid Freeform Fabr Symp*.

Wang, L., Ran, X., Li, Y., Li, F., Liu, J., Du, J., Zhang, X., and Qi, X. (2020). Energy consumption model of plasma spraying based on unit process life cycle inventory. *J of Mater Res and Tech*, 9(6):15324–15334. https://doi.org/10.1016/j.jmrt.2020.11.007.

Wernet, G., Bauer, C., Steubing, B., Reinhard, J., Moreno-Ruiz, E., and Weidema, B. (2016). The ecoinvent database version 3 (part i): overview and methodology. *Int J of Life Cycle Assess*, 21(9):1218–1230. https://doi.org/10.1007/s11367-016-1087-8.
